# Combined APRI/ALBI score to predict mortality after hepatic resection

**DOI:** 10.1093/bjsopen/zraa043

**Published:** 2021-01-17

**Authors:** P Starlinger, D S Ubl, H Hackl, J Starlinger, D M Nagorney, R L Smoot, E B Habermann, S P Cleary

**Affiliations:** Department of Surgery, Division of Hepatobiliary and Pancreas Surgery, Mayo Clinic, Rochester, Minnesota, USA; Mayo Clinic Robert D and Patricia E Kern Center for the Science of Health Care Delivery and Department of Health Services Research, Mayo Clinic, Rochester, Minnesota, USA; Division of Bioinformatics, Biocenter, Medical University of Innsbruck, Innsbruck, Austria; 37binary UG, Berlin, Germany; Department of Surgery, Division of Hepatobiliary and Pancreas Surgery, Mayo Clinic, Rochester, Minnesota, USA; Department of Surgery, Division of Hepatobiliary and Pancreas Surgery, Mayo Clinic, Rochester, Minnesota, USA; Mayo Clinic Robert D and Patricia E Kern Center for the Science of Health Care Delivery and Department of Health Services Research, Mayo Clinic, Rochester, Minnesota, USA; Department of Surgery, Division of Hepatobiliary and Pancreas Surgery, Mayo Clinic, Rochester, Minnesota, USA

## Abstract

**Background:**

Aspartate aminotransferase/platelet ratio index (APRI) and albumin–bilirubin grade (ALBI) are validated prognostic indices implicated as predictors of postoperative liver dysfunction after hepatic resection. The aim of this study was to evaluate the relevance of the combined APRI/ALBI score for postoperative clinically meaningful outcomes.

**Methods:**

Patients undergoing hepatectomy were included from the American College of Surgeons National Surgical Quality Improvement Program database. The association between APRI/ALBI score and postoperative grade C liver dysfunction, liver dysfunction-associated and overall 30-day mortality was assessed.

**Results:**

A total of 12 055 patients undergoing hepatic resection from 2014 to 2017 with preoperative blood values and detailed 30-day postoperative outcomes were included (exploration cohort: January 2014 to December 2016; validation cohort: 2017). In the exploration cohort (8538 patients), the combination of both scores (APRI/ALBI) was significantly associated with postoperative grade C liver dysfunction, 30-day mortality, and liver dysfunction-associated 30-day mortality, and was superior to either score alone. The association with postoperative 30-day mortality was confirmed in multivariable analysis. A predictive model was generated using the exploration cohort. The predicted incidence of events closely followed the observed incidence in the validation cohort (3517 patients). Subgroup analyses of tumour types were used to generate disease-specific risk models to assess risk in different clinical scenarios. These findings informed development of a smartphone application (https://tellaprialbi.37binary.com).

**Conclusion:**

The predictive potential of the combined APRI/ALBI score for clinically relevant outcomes such as mortality was demonstrated. An evidence-based smartphone application will allow clinical translation and facilitation of risk assessment before hepatic resection using routine laboratory parameters.

## Teaser

Preoperative risk assessment before liver surgery remains challenging. This study documented and confirmed that the combination of two scores (aspartate aminotransferase/platelet ratio index (APRI) and albumin–bilirubin grade (ALBI)) using readily available blood tests allows precise and reliable identification of patients at highest risk of postoperative death. Designing a smartphone application will allow easy application of these results, and could significantly improve patient care by identifying patients who might be at highest operative risk and therefore better served by other treatment strategies

## Resumen

**Antecedentes:**

El índice de relación aspartato aminotransferasa/plaquetas (*aspartate aminotransferase/platelet ratio index*, APRI) y grado de albúmina-bilirrubina (albumin-bilirubin grade, ALBI) son índices pronóstico validados implicados como predictores de disfunción hepática postoperatoria (*liver dysfunction*, LD) después de una resección hepática. El objetivo de este estudio fue evaluar la relevancia de la puntuación combinada APRI/ALBI respecto los resultados postoperatorios clínicamente significativos.

**Métodos:**

Los pacientes sometidos a hepatectomía se incluyeron en la base de datos del *American College of Surgeons National Surgical Quality Improvement Program* (NSQIP). Se evaluó la asociación de APRI/LBI con LD grado C postoperatorio, así como con LD asociada y mortalidad global a los 30 días.

**Resultados:**

Se incluyeron 12.055 pacientes sometidos a resección hepática entre 2014 y 2017 con valores sanguíneos preoperatorios y resultados postoperatorios detallados a los 30 días (cohorte de exploración: 01/2014-12/2016; cohorte de validación: 2017). En la cohorte de exploración (*n* = 8.538), la combinación de ambas puntuaciones (APRI/ALBI) se asoció significativamente con LD grado C postoperatorio, mortalidad a 30 días, mortalidad asociada a LD a los 30 días y fue superior a cualquiera de las puntuaciones por separado. La asociación con la mortalidad postoperatoria a los 30 días se confirmó mediante el análisis multivariable. Se generó un modelo predictivo utilizando la cohorte de exploración. La incidencia anticipada de eventos se ajustó a la incidencia observada en la cohorte de validación (*n* = 3.517). Se utilizaron análisis de subgrupos de tipos de tumores a fin de generar modelos de riesgo específicos de la enfermedad para evaluar el riesgo en diferentes escenarios clínicos. Estos hallazgos permitieron el desarrollo de una aplicación para teléfonos inteligentes (https://tellaprialbi.37binary.com).

**Conclusión:**

Se demostró el potencial predictivo de la puntuación combinada APRI/ALBI para parámetros de resultados clínicamente relevantes, tales como la mortalidad. Una aplicación de teléfono inteligente basada en la evidencia permite la traducción clínica y facilita la evaluación de riesgos antes de la resección hepática utilizando parámetros de laboratorio de rutina.

## Introduction

Preoperative assessment of postoperative risk remains a major challenge in patients undergoing hepatic resection. A major cause of persistent difficulties in preoperative assessment of risk of postoperative death is the enormous heterogeneity in liver function in patients undergoing hepatic surgery. In particular, in patients with hepatocellular carcinoma (HCC), underlying liver disease can range from marginally compensated cirrhosis to completely unaffected healthy liver parenchyma. Additionally, neoadjuvant chemotherapy, which is now frequently used in patients with metastatic disease to the liver^1–3^, can negatively affect liver function by causing chemotherapy-associated liver injury (CALI)^4^. The degree of CALI varies significantly among patients, ranging from steatosis, sinusoidal obstruction syndrome to chemotherapy-associated steatohepatitis^5^. Similarly, the growing incidence of non-alcoholic steatohepatitis and associated non-alcoholic fatty liver disease poses another frequent source of underlying liver disease, affecting postoperative outcome after hepatic resection^6^^,^^7^. The quantification of these differences in terms of hepatic function remains difficult. Aspartate to platelet ratio index (APRI) and albumin–bilirubin grade (ALBI) scores were shown as estimates of liver function similar to the Child–Pugh score and indocyanine green (ICG) clearance^8–15^. Recently, the combination of APRI and ALBI scores was shown to be predictive of postoperative outcome after hepatic resection in patients with metastatic colorectal cancer to the liver, and correlated dynamically with the recovery of CALI after cessation of chemotherapy, offering the potential for optimized timing of surgery^16^^,^^17^. Similarly, the combination of APRI and ALBI scores was predictive of outcome after hepatic resection in patients with HCC, and the combination of these scores was superior to each score alone^18^.

The aim of this study was to assess the predictive potential of the combination of APRI and ALBI scores in patients undergoing hepatic resection for a variety of indications in a large cohort of patients undergoing hepatic resection documented in the American College of Surgeons procedure-targeted National Surgical Quality Improvement Program (NSQIP) database on hepatectomy.

## Methods

The procedure-targeted NSQIP database on hepatectomy contains prospectively collected information on patients undergoing hepatic resections in several institutions in the USA. Basic characteristics, preoperative laboratory values, and preoperative co-morbidities are documented routinely.

All patients who underwent hepatic resection between 1 January 2014 and 31 December 2017 were included from the procedure-targeted NSQIP database on hepatectomy using current procedural terminology codes: partial hepatectomy (47120) (defined as minor hepatectomy); trisegmentectomy (47122), left lobectomy (47125), and right lobectomy (47130) (defined as major hepatectomy). Patients aged less than 18 years and those with trauma were excluded from the final analysis. The patients were divided into an exploration cohort including patients from the years 2014–2016, and a validation cohort including patients from 2017. Patients were further grouped according to underlying neoplastic entity for subgroup analyses.

Morbidity was assessed within 30 days after surgery, and divided into major and minor complications. Minor complications constituted superficial surgical-site infection (SSI), urinary tract infection, deep incisional SSI, organ space SSI, wound disruption, sepsis, progressive renal insufficiency, deep vein thrombosis or thrombophlebitis. Pulmonary embolism, cardiac arrest requiring cardiopulmonary resuscitation, myocardial infarction, stroke/cerebrovascular accident with neurological deficit, unplanned intubation, ventilator requirement for more than 48 h, pneumonia, acute renal failure, and return to the operating room were defined as major complications.

### Calculation of liver dysfunction scores

Both, APRI and ALBI were calculated in accordance with previously published formulas (*Fig. S1*)^16^. The Model for End-Stage Liver Disease (MELD) score was calculated using the standard equation: MELD score=(0.957 × ln[creatinine, mg/dl]+0.378 × ln[total bilirubin, mg/dl]+1.120 × ln[INR]+0.643)×10), where INR is the international normalized ratio. As the timing of dialysis was not documented routinely in the database, patients who were recorded as undergoing chronic dialysis were excluded from comparative analyses including the MELD score.

**Fig. 1:**
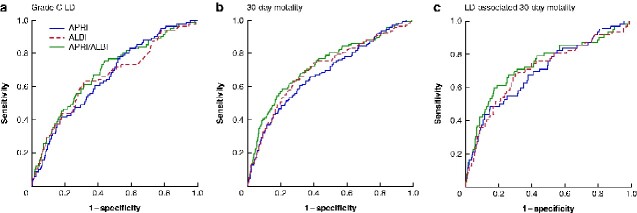
Receiver operating characteristic (ROC) curves comparing predictive ability of APRI and ALBI scores, individually or in combination, in relation to clinical outcomes after hepatic resection **a** Postoperative grade C liver dysfunction (LD), **b** postoperative 30-day mortality, and **c** LD-associated 30-day mortality. Areas under the curve: **a** aspartate aminotransferase/platelet ratio index (APRI) 0.662, albumin–bilirubin grade (ALBI) 0.659, APRI/ALBI 0.689; **b** APRI 0.679, ALBI 0.704, APRI/ALBI 0.728; **c** APRI 0.697, ALBI 0.704, APRI/ALBI 0.735.

### Definition and classification of outcome parameters

The procedure-targeted NSQIP database on hepatectomy contains detailed prospective data on postoperative outcome in terms of postoperative liver dysfunction (LD), mortality; the data are of very high quality. In particular, the severity of postoperative LD is further classified according to the International Study Group of Liver Surgery (ISGLS) criteria^19^: grade A, abnormality in laboratory parameters not requiring a change in clinical management; grade B, deviation from clinical management without invasive treatment; and grade C, deviation from clinical management requiring invasive treatment. Postoperative LD-associated mortality was recorded for patients who died within 30 days and concomitantly fulfilled any ISGLS grade LD. Postoperative morbidity was defined as the development of one or more postoperative complications. The postoperative observation period was 30 days, as in the NSQIP database.

### Statistical analysis

Statistical analyses were based on parametric tests and *P* < 0.050 was considered statistically significant. Receiver operating characteristic (ROC) curve analysis was used to assess and compare the predictive potential of APRI, ALBI, and their combination for postoperative grade C LD, 30-day mortality, and LD-associated mortality. Ultimately, a multivariable model was fit, based on logistic regression including all variables significant in univariable analysis. To determine whether the APRI/ALBI score as a continuous variable had a non-linear effect on the risk of 30-day mortality, generalized additive models with smoothing splines were applied using R version 3.6.1 package mgcv (R Foundation for Statistical Computing, Vienna, Austria,). Log odds with 95 per cent c.i. (without intercept/baseline from the model) and average predicted individual risk (percentage probability probability) were illustrated over the range of APRI/ALBI scores (grouped in deciles). Statistical analyses were performed using SPSS^®^ version 23 (IBM, Armonk, New York, USA).

## Results

A total of 13 401 patients underwent elective hepatic resection between January 2014 and December 2017. Of these, 1305 patients underwent concomitant abdominal procedures and were therefore excluded. A further 41 patients with obvious documentation errors were also excluded from analyses. To allow validation, the cohort was divided into an exploration/model generation cohort (including 8538 patients who had surgery between April 2014 and December 2016) and a validation cohort (3517 patients operated in 2017). Characteristics of the two cohorts are summarized in *Table 1*.

**Table 1:** Patient demographics

	Evaluation cohort (*n* = 8538)	Validation cohort (*n* = 3517)
**Baseline characteristics**		
Sex ratio (M : F)	4245 : 4293	1812 : 1705
Neoplastic entity		
Metastatic disease to liver	3778 (44.2)	1449 (41.2)
HCC	1552 (18.2)	719 (20.4)
CCA	937 (11.0)	406 (11.5)
Perihilar CCA	206 (2.4)	72 (2.0)
Benign	874 (10.2)	395 (11.2)
Other	1190 (13.9)	476 (13.5)
Co-morbidities		
ASA grade		
I	186 (2.2)	55 (1.6)
II	2237 (26.2)	909 (25.8)
III	5529 (64.8)	2313 (65.8)
IV	565 (6.6)	240 (6.8)
Unknown	21 (< 0.1%)	0 (0.0%)
Diabetes	1425 (16.7)	679 (19.3)
Smoking	1315 (15.4)	566 (16.1)
BMI (kg/m^2^)*	27.52 (12.6–89.46)	27.71 (15.18–64.41)
Dialysis	16 (0.2)	6 (0.2)
Preoperative laboratory measurements*		
Platelets (× 10^3^/µl)	219 (24–861)	218 (46–708)
Serum bilirubin (mg/dl)	0.5 (0.1–14.8)	0.5 (0.1–14.0)
AST (units/l)	26 (3–841)	26 (3–815)
Albumin (g/dl)	4.1 (1.3–9.7)	4.0 (1.4–7.4)
International normalized ratio	1.0 (0.1–10.0)	1.4 (0.8–10.0)
Creatinine (mg/dl)	0.8 (0.1–10.3)	0.8 (0.3–14.2)
APRI score	0.240 (0.019–8.894)	0.246 (0.034–7.477)
ALBI score	–2.785 (–7.431 to 1.376)	–2.773 (–5.623 to 1.164)
APRI/ALBI score	–2.543 (–7.352 to 7.318)	–2.539 (–5.032 to 5.809)
MELD score	7 (6–32)	7 (6–32)
**Operative data**		
Hepatic resection		
Minor	5219 (61.1)	2159 (61.4)
Major	3319 (38.9)	1358 (38.6)
Laparoscopic/robotic^†^	2255 (26.4)	936 (26.6)
Duration of surgery (min)*	212 (6–1027)	203 (5–758)
Hepatectomy with bile duct resection	427 (5.0)	141 (4.0)
Hepatectomy with vascular resection	205 (2.4)	76 (2.2)
**Postoperative outcomes**		
Readmission	763 (8.9)	306 (8.7)
30-day mortality	137 (1.6)	51 (1.5)
LD-associated 30-day mortality	76 (0.9)	31 (0.9)
Duration of postoperative hospital stay (days)*	5 (0–119)	5 (0 – 85)
ISGLS LD grade		
No LD	8132 (95.2)	3383 (96.2)
Grade A	173 (2.0)	55 (1.6)
Grade B	137 (1.6)	41 (1.2)
Grade C	96 (1.1)	38 (1.1)
Readmission	763 (8.9)	306 (8.7)
Morbidity, any	1528 (17.9)	604 (17.2)
Morbidity, major	1257 (14.7)	458 (13.0)
Unplanned reintubation	175 (2.0)	57 (1.6)
Reoperation	205 (2.4)	90 (2.6)
Sepsis	430 (5.0)	137 (3.9)
Ventilation for > 48 h	132 (1,5)	56 (1.6)
Pneumonia	268 (3.1)	86 (2.4)

Values in parentheses are percentages unless indicated otherwise; *values are median (range). ^†^Including laparoscopic attempts with conversion. HCC, hepatocellular carcinoma; CCA, cholangiocarcinoma; AST, aspartate aminotransferase; APRI, aspartate aminotransferase/platelet ratio index; ALBI, albumin–bilirubin grade; MELD, Model for End-Stage Liver Disease; LD, liver dysfunction; ISGLS, International Study Group of Liver Surgery.

### Association between ALBI, APRI, APRI/ALBI and postoperative complications

The predictive potential of preoperative APRI and ALBI for postoperative grade C LD, 30-day mortality, and LD-associated 30-day mortality was assessed in the exploration cohort. Patients who developed postoperative grade C LD had higher APRI (*Fig. S2a*) and ALBI (*Fig. S2d*) scores before hepatic resection. Postoperative 30-day mortality (not significant) and LD-associated 30-day mortality were associated with higher preoperative APRI (*Fig. S2b,c*) and ALBI (*Fig. S2e,f*) scores. The combined APRI/ALBI score was significantly higher in patients with postoperative grade C LD, 30-day mortality, and LD-associated 30-day mortality (*Fig. S2g–i*).

**Fig. 2:**
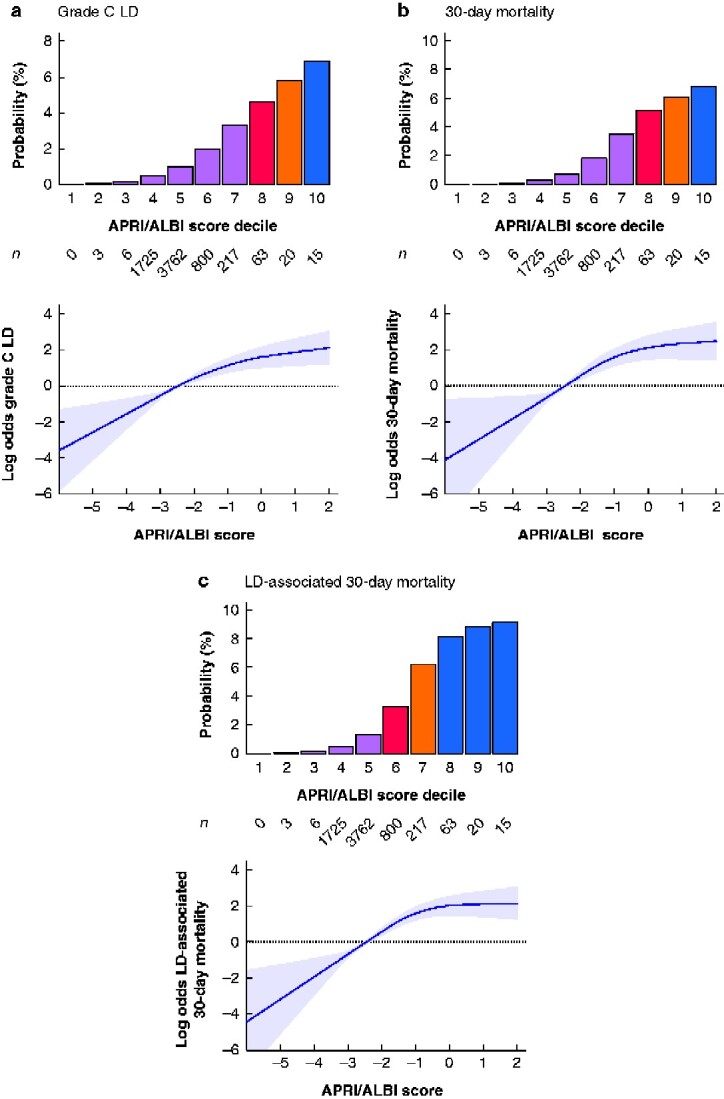
Model generation for individualized prediction of postoperative outcome Given the continuous non-linear relationship between combined aspartate aminotransferase/platelet ratio index/albumin–bilirubin grade (APRI/ALBI) score and clinical outcomes, generalized additive models with smoothing splines were used, as illustrated for **a** postoperative grade C liver dysfunction (LD), **b** postoperative 30-day mortality, and **c** LD-associated 30-day mortality. Shaded areas represent 95 per cent confidence intervals. Based on the respective model, the combined APRI/ALBI score was grouped into deciles and individual risk for each outcome is shown in the lower panels. Number of patients in each decile is also shown.

To avoid overfitting, a previously published cut-off value for the combined APRI/ALBI score of –2.46^16^ was used to define a high and a low APRI/ALBI group in the exploratory cohort. Rates of various postoperative outcomes documented routinely in the procedure-targeted NSQIP database on hepatectomy were compared. Accordingly, a consistent increase in multiple postoperative outcomes in patients with an increased APRI/ALBI score was found (*Fig. S3*).

**Fig. 3:**
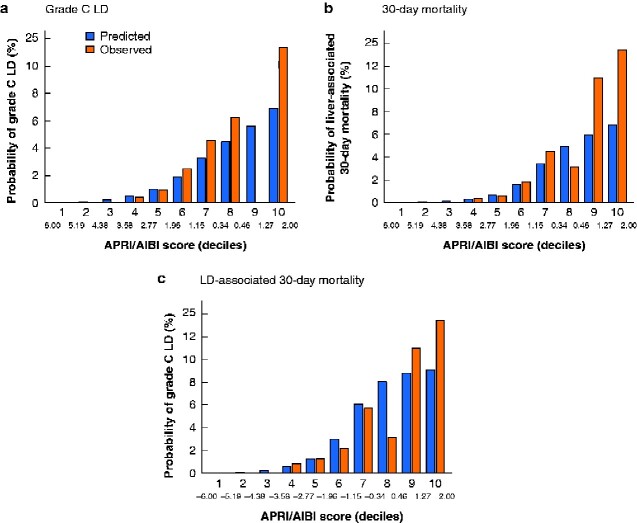
Assessment of prediction models in validation cohort Model-based predicted incidence by aminotransferase/platelet ratio index/albumin–bilirubin grade (APRI/ALBI) score decile of **a** postoperative grade C liver dysfunction LD, **b** postoperative 30-day mortality, and **c** LD-associated 30-day mortality, compared with actual incidence in the validation cohort.

To compare the discriminatory potential of the APRI and ALBI scores individually and in combination for the clinically relevant outcomes grade C LD, 30-day mortality, and LD-associated 30-day mortality, ROC curve analyses were undertaken in the exploration cohort. ALBI and APRI alone appeared to be equally effective in their predictive potential for grade C LD and LD-associated 30-day mortality, whereas ALBI seemed to be slightly superior for 30-day mortality (*Fig. 1*). The combined APRI/ALBI score exceeded the predictive potential of the individual scores.

A multivariable analysis was performed to test whether the combined APRI/ALBI score was an independent predictor of 30-day mortality, and to identify potential confounding factors (*Table 2*). Combined APRI/ALBI score, MELD score, sex, age, ASA grade, diabetes, extent of resection, duration of surgery, and preoperative albumin, bilirubin, aspartate aminotransferase and platelet levels showed an association with 30-day mortality in univariable analysis, and were entered into the multivariable model. In multivariable analysis, only the combined APRI/ALBI score, sex, age, diabetes, extent of resection, and duration of surgery remained as significant, independent variables (*Table 2*).

**Table 2:** Results of univariable and multivariable logistic regression analyses to identify predictors of postoperative 30-day mortality

	Univariable analysis		Multivariable analysis	
	Odds ratio	*P*	Odds ratio	*P*
**Preoperative scores**				
APRI/ALBI	1.73 (1.52, 1.96)	< 0.001	1.72 (1.16, 2.55)	0.007
MELD	1.14 (1.09, 1.20)	< 0.001	1.04 (0.95, 1.13)	0.411
**Patient characteristics**				
Male sex	0.36 (0.25, 0.53)	< 0.001	0.59 (0.35, 1.00)	0.049
Age (years)	1.06 (1.05, 1.08)	< 0.001	1.06 (1.04, 1.09)	< 0.001
BMI (kg/m^2^)	1.01 (0.99, 1.04)	0.303		
ASA grade	3.04 (2.23, 4.14)	< 0.001	1.45 (0.92, 2.28)	0.114
Neoplastic entity	0.98 (0.90, 1.07)	0.653		
Race	0.98 (0.80, 1.19)	0.809		
Smoking	1.01 (0.63, 1.61)	0.981		
Diabetes	2.84 (2.00, 4.05)	< 0.001	1.76 (1.08, 2.87)	0.024
**Operative data**				
Major resection	3.39 (2.36, 4.87)	< 0.001	2.14 (1.28, 3.59)	0.004
Duration of surgery (min)	1.006 (1.005, 1.007)	< 0.001	1.005 (1.004, 1.007)	< 0.001
**Preoperative laboratory measurements**				
Albumin (g/dl)	0.34 (0.25, 0.46)	< 0.001	0.96 (0.53, 1.75)	0.903
Serum bilirubin (mg/dl)	1.32 (1.20, 1.46)	< 0.001	0.95 (0.79, 1.15)	0.611
AST (units/l)	1.008 (1.005, 1.010)	< 0.001	0.997 (0.990, 1.005)	0.447
Platelets (× 10^3^/µl)	0.998 (0.996, 1.000)	0.049	0.999 (0.996, 1.002)	0.393

Values in parentheses are 95 per cent confidence intervals. APRI, aspartate aminotransferase/platelet ratio index; ALBI, albumin–bilirubin grade; MELD, Model for End-Stage Liver Disease; AST, aspartate aminotransferase. Odds ratios for continuous variables are shown per unit increase.

### Generation of an individual-patient risk stratification model

To evaluate a continuous non-linear relationship between the combined APRI/ALBI score and risk of grade C LD, 30-day mortality or LD-associated 30-day mortality, generalized additive models with smoothing splines were applied to the exploratory cohort (*Fig. 2*). To allow better visualization and categorization, the APRI/ALBI score was further divided according to decile risk groups, to facilitate reliable risk stratification for each patient. Although the majority of patients were in the low–intermediate-risk group for these outcomes, a relevant number of patients were at significantly increased risk of postoperative death (*Fig. 2*, lower panels).

### Validation of preoperative APRI/ALBI score-based prediction model

Given the significant risk of possible overfitting, the initial results were validated in an independent validation cohort of 3517 patients who had surgery in 2017. When the cohort was divided into risk groups according to deciles of APRI/ALBI score, a close correlation between predicted and observed outcomes in terms of grade C LD, 30-day mortality, and LD-associated 30-day mortality in the validation cohort was found, with rates that closely followed the predictive model across the risk groups (*Fig. 3*).

### Predictive potential of APRI/ALBI score compared with MELD score

**Fig. 4:**
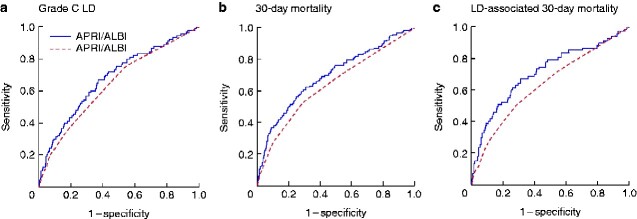
Receiver operating characteristic (ROC) curves comparing predictive power of combined APRI/ALBI score and MELD score for postoperative outcomes **a** Postoperative grade C liver dysfunction (LD), **b** postoperative 30-day mortality, and **c** LD-associated 30-day mortality. Areas under the curve: **a** aspartate aminotransferase/platelet ratio index (APRI) 0.640, albumin–bilirubin grade (ALBI) 0.655, APRI/ALBI 0.678, Model for End-Stage Liver Disease (MELD) 0.637; **b** APRI 0.647, ALBI 0.684, APRI/ALBI 0.700, MELD 0.633; **c** APRI, 0.651, ALBI 0.689, APRI/ALBI 0.710; MELD 0.630. For clarity, only curves for APRI/ALBI and MELD scores are shown.

The predictive value of the APRI and ALBI scores individually and combined was compared with that of the MELD score, a current widely used and validated predictor of risk after hepatic resection. The three outcomes (grade C LD, 30-day mortality, and LD-associated 30-day mortality) were assessed using ROC curve analyses. Although all scores were highly significantly associated with each clinical outcome variable (*P* < 0.001), the MELD score seemed inferior to the individual APRI and ALBI scores in its predictive value (*Fig. 4*). The combined APRI/ALBI score exceeded the predictive value of MELD for each outcome.

### Subgroup analyses for risk assessment in specified patient cohorts.

**Fig. 5:**
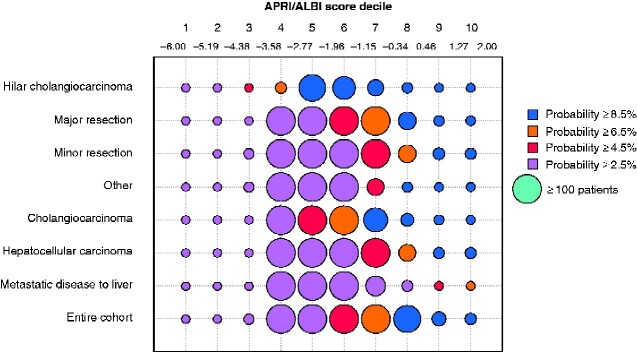
Bubble plot illustrating association between combined APRI/ALBI score and postoperative 30-day mortality in patient subgroups Incidence and individual risk of postoperative 30-day mortality (percentage probability) is shown in relation to combined aspartate aminotransferase/platelet ratio index/albumin–bilirubin grade (APRI/ALBI) score deciles for several patient and surgical subgroups. The change from green to red indicates a gradual increase in risk, with red indicating a higher risk of postoperative 30-day mortality. Increased bubble size reflects large number of patients.

**Fig. 6:**
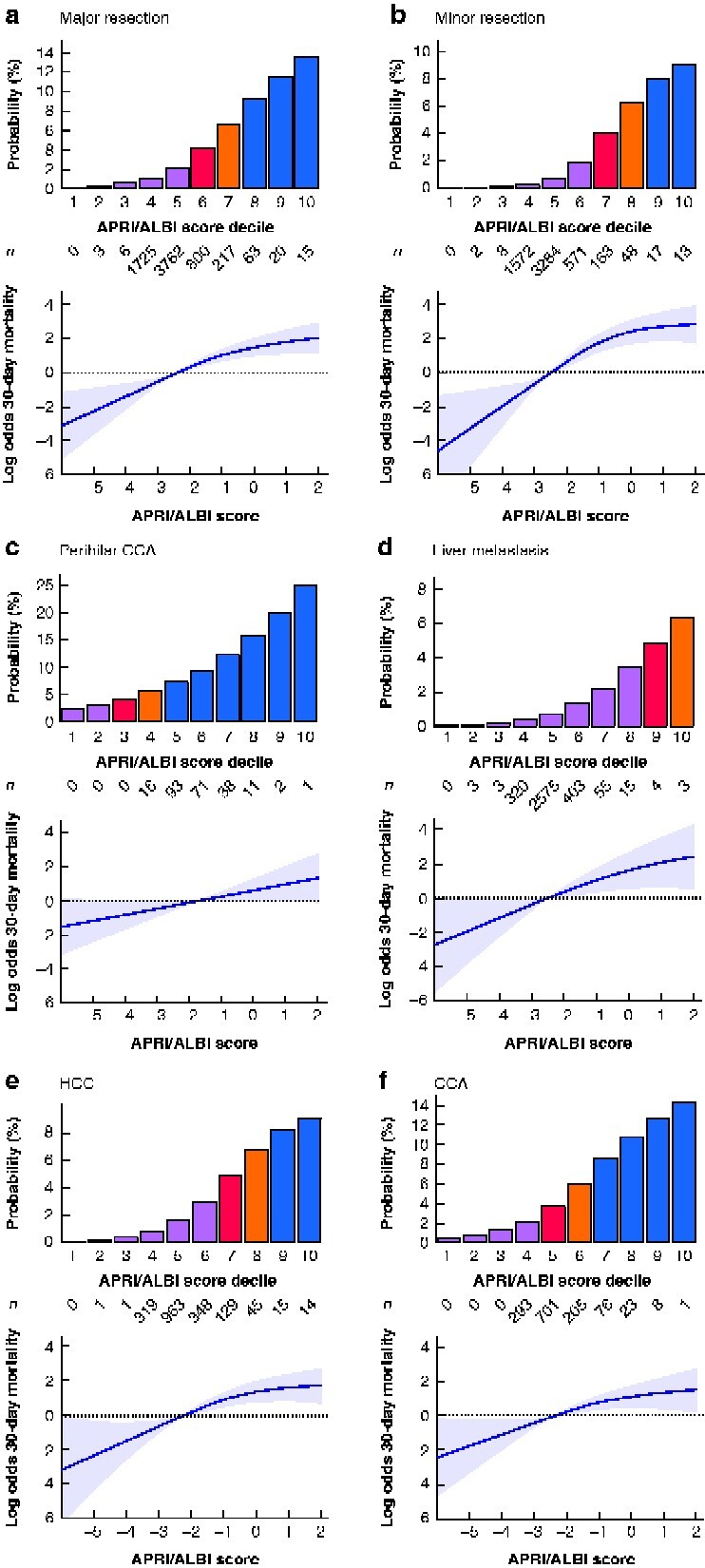
Subgroup model generation for individualized prediction of postoperative 30-day mortality Generalized additive models with smoothing splines were used, as illustrated for postoperative 30-day mortality in patient subgroups: **a** major resection, **b** minor resection, **c** perihilar cholangiocarcinoma (CCA), **d** metastatic disease to the liver, **e** hepatocellular carcinoma (HCC), and **f** CCA. Shaded areas represent 95 per cent confidence intervals. Based on the respective model, the combined aspartate aminotransferase/platelet ratio index/albumin–bilirubin grade (APRI/ALBI) score was grouped into deciles and individual risk for each subgroup is shown in the lower panel. Number of patients in each decile is also shown.

As different disease patterns, surgical indications, and extent of resection are associated with differences in risk, patients were divided according to tumour type as well as extent of resection. Despite a uniform predictive value of the combined APRI/ALBI score, distinct shifts in risk scores were observed in individual patient subgroups (*Fig. 5*). To optimize and individualize risk prediction within patient subgroups, subgroup-specific prediction models were generated to allow the highest precision in outcome prediction (*Fig. 6*). Using these results, a smartphone application was designed to allow specific selection of subgroups for personalized individual-patient risk assessment (https://tellaprialbi.37binary.com).

## Discussion

Postoperative severe LD and mortality remain critical issues in patients undergoing hepatic resection. Here, the predictive value of the combination of two commonly used liver function scores, APRI and ALBI, was explored in a representative cohort of 12 055 patients undergoing hepatic resection. The combined APRI/ALBI score was found to be specifically predictive of clinically significant poor postoperative outcomes: LD requiring intervention (grade C), overall 30-day mortality, and LD-associated 30-day mortality. APRI/ALBI score was a better predictor of these outcomes than the widely used and validated MELD score. Furthermore, the predictive value of the combined score was verified in patients with different neoplasms and different hepatic resections. The large size of the NSQIP database allowed the development of risk prediction models for disease- and operation-specific subgroups to enhance the precision of risk assessment. Finally, a smartphone application was developed to calculate individual-patient risk for postoperative 30-day mortality that is easily applicable to the clinical setting (https://tellaprialbi.37binary.com).

The severity of liver disease varies substantially in patients undergoing hepatic resection, posing a challenge for individualized care in these patients. HCC has been the best studied malignancy for preoperative risk assessment. Indeed, the recent American Association for the Study of Liver Diseases (AASLD^20^)/European Association for the Study of the Liver (EASL^21^) international guidelines for HCC state that patients with portal hypertension should not undergo hepatic resection. However, quantification of portal hypertension requires invasive measurements, and clinical surrogates may not reflect the severity of portal hypertension accurately which may confound indication for resection. Moreover, previous reports documented good results of surgery even in patients with portal hypertension^22^. Several invasive and non-invasive tests have been developed to assess preoperative risk for postoperative outcome after hepatic resection, but few have been incorporated into routine clinical practice even in high-risk patients. Major drawbacks of currently used predictors are availability, high costs, and invasiveness^23^. Although hepatic venous pressure gradient is predictive of postoperative clinical outcome in patients with HCC^24–26^, it remains in limited use because of the invasive nature of the test. Other less invasive and well established markers to assess liver function rely on dynamic functional assessment of the liver. Multiple groups have documented that ICG clearance is predictive of postoperative LD and morbidity^27^; however, ICG clearance testing and most other measures of hepatic function are expensive and time-consuming. The APRI/ALBI score uses commonly assessed laboratory tests, and represents a simple non-invasive, and easily accessible risk stratification method.

The increased performance of the combined APRI and ALBI score and its broad applicability to different patient subgroups likely reflect differences between components of the score for assessing various pathophysiological backgrounds. Indeed, ALBI seems to correlate more strongly with advanced fibrosis, whereas APRI score is better associated with CALI. Although advanced fibrosis and cirrhosis pose a significant risk for postoperative fatal outcome after hepatic resection, the relevance of CALI is often underestimated2^28^. ICG clearance^29^^,^^30^ has been used for identification of patients with CALI, but to date has not been employed widely and routinely. Liver biopsy clearly provides the most accurate method for diagnosis of CALI. However, percutaneous biopsy is reserved for selected patients with high suspicion of severe liver damage after chemotherapy, which leaves the majority of patients without adequate preoperative assessment for CALI. Moreover, liver biopsy and histopathological examination have low sensitivity for most types of CALI3^31^. The APRI/ALBI score might not only detect underlying liver disease from other causes, but also pick up CALI and thereby be used to optimize preoperative risk stratification in these patients, as clearly suggested by the present results. An interesting aspect of previous findings was that the combined APRI/ALBI score changed dynamically during neoadjuvant chemotherapy, suggesting a direct correlation between the marker and induced liver damage^16^. Indeed, this finding is in line with recent reports of the amelioration of CALI after chemotherapy cessation^32^. This suggests that repetitive assessment of the APRI/ALBI score after neoadjuvant chemotherapy might be an attractive tool to define an optimized time point for liver surgery in these patients.

A limitation of this study is the retrospective nature of the analyses. However, assessing clinically relevant outcome parameters such as mortality in a prospective fashion remains a major challenge given the low incidence. Another limitation is that the number of patients in the highest risk groups is low. However, included patients were all deemed fit for surgery and therefore had been preselected. In clinical daily life, patients in these risk groups might therefore be significantly more common. Ultimately, the gradually increasing risk per group should be used to stratify patients with respect to patient-related factors such as co-morbidities and extent of surgery to allow individualized risk assessment for lower-risk groups as well.

*Disclosure.* The authors declare no conflicts of interest.

## Supplementary material

Supplementary material is available at *BJS Open* online.

## Supplementary Material

zraa043_Supplementary_Data
