# Supplementary material for: Combined APRI/ALBI score to predict mortality after hepatic resection
Source: BJS Open. 2021 Jan 17;5(1):zraa043. doi: 10.1093/bjsopen/zraa043 (PMC7893465; doi:10.1093/bjsopen/zraa043)

**Fig. S1 ALRP/ALBI Score calculation formula.**

**
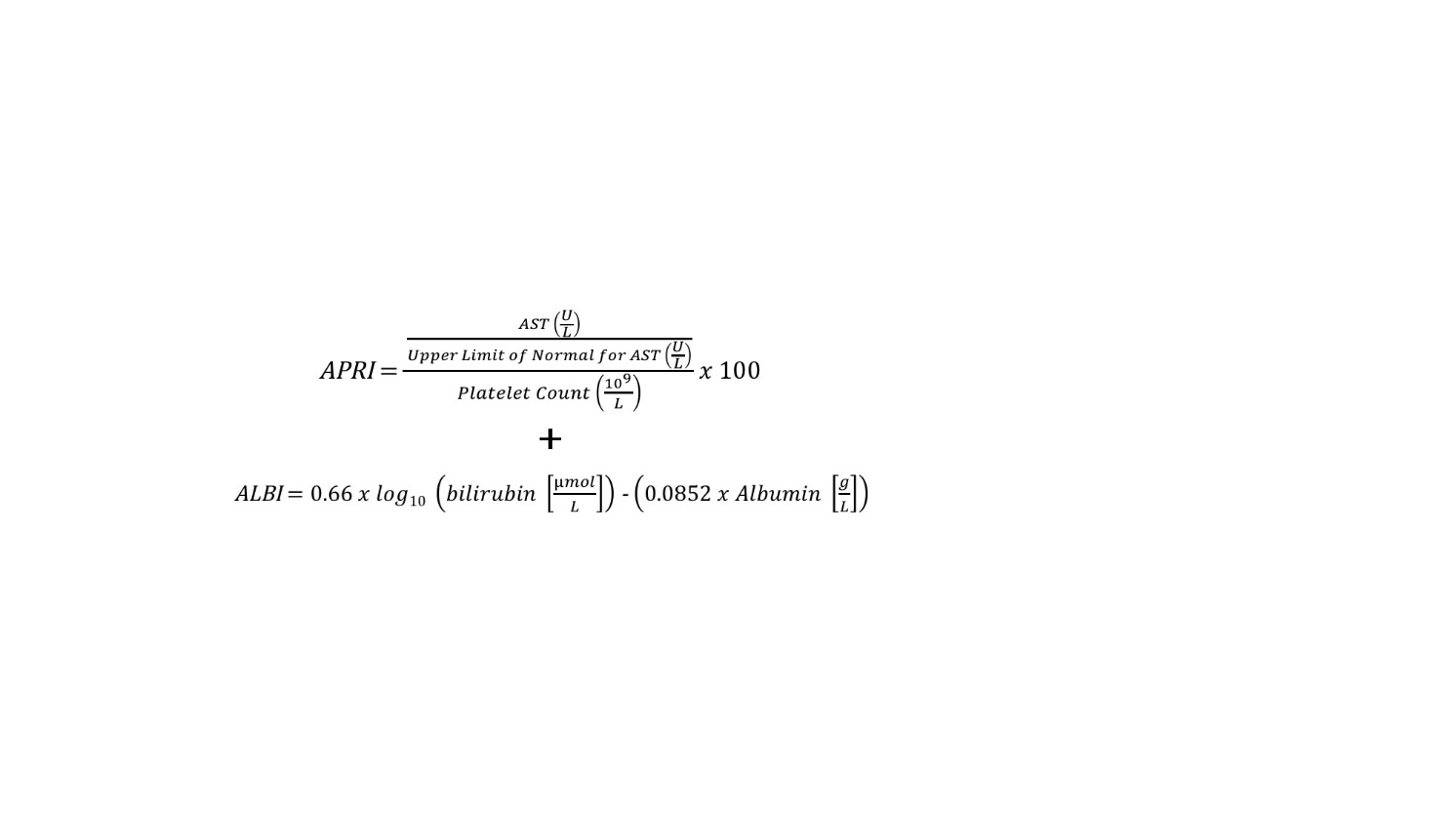
**

**Fig. S2** **ALBI, APRI and APRI/ALBI in accordance to clinical outcome after hepatic resection**

Levels of APRI (A,B,C), ALBI (D,E,F) and APRI/ALBI (G,H,I) are shown for patients with or without postoperative liver dysfunction (LD) Grade C (A,D,G), for postoperative with 30-day mortality (mort.) (B,E,H) and for patients with or without postoperative liver dysfunction associated (assoc.) mortality (C,F,I). Boxplots are given without outliers to improve resolution of interquartile ranges. *P<0.0001

**
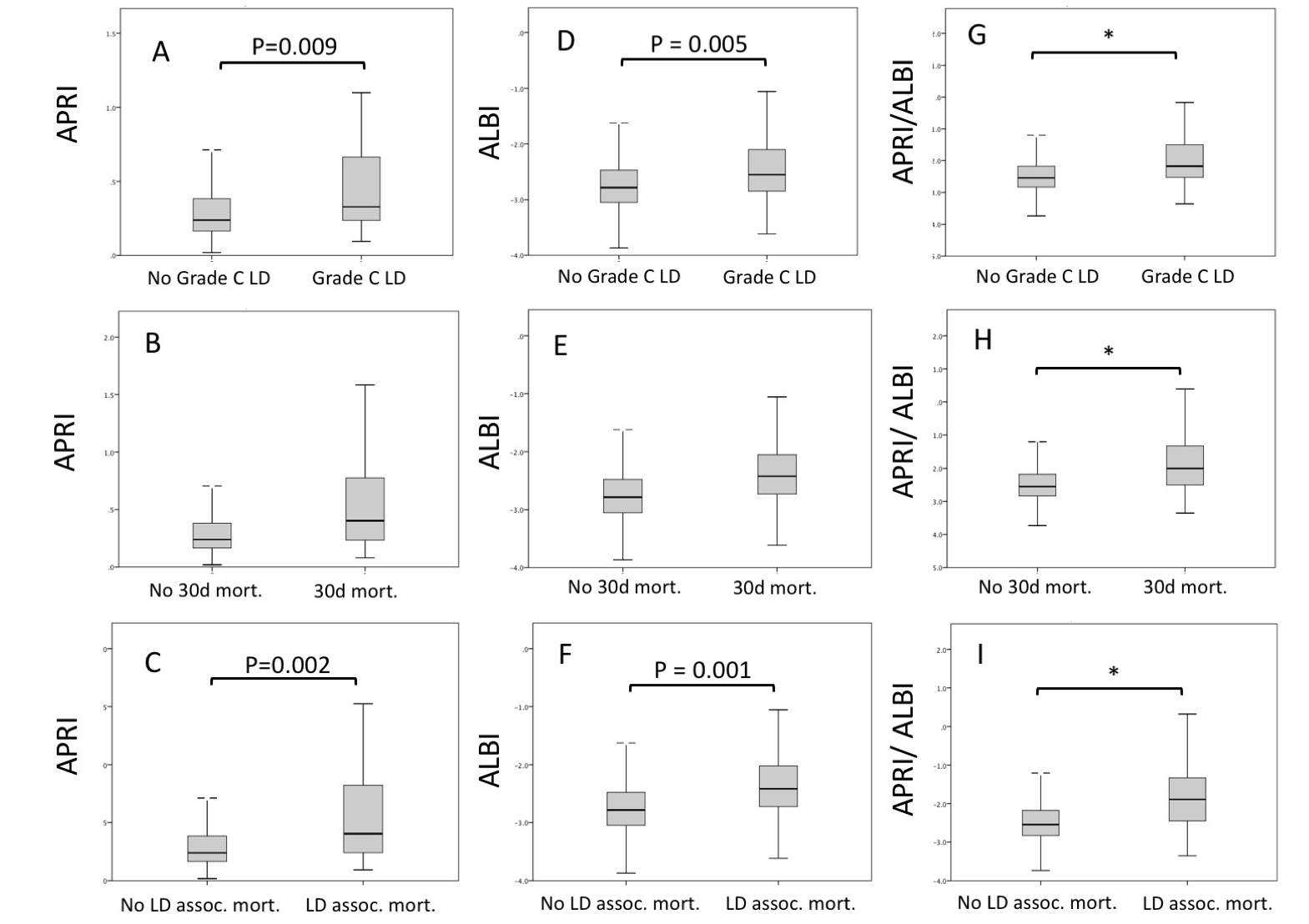
**

**Fig. S3 Patients above the cut-off at -2.46 of APRI/ALBI show higher incidence of a wide variety of postoperative complications.**

Patients were divided according to the recently defined cut-off. Postoperative complication rates are illustrated in percentage per group. *P<0.0001


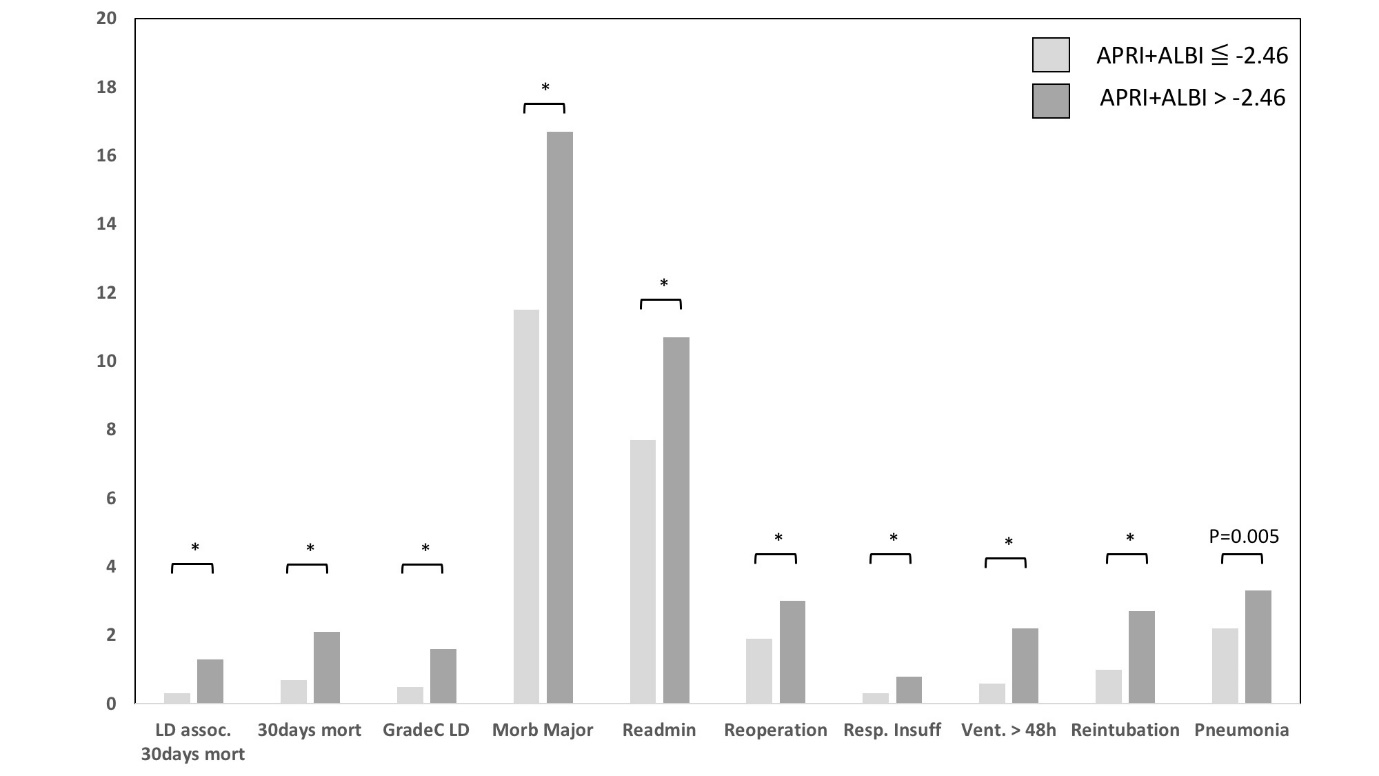

Supplement: zraa043_Supplementary_Data [file zraa043_supplementary_data.docx]
